# A Machine Learning Model Utilizing a Novel SNP Shows Enhanced Prediction of Coronary Artery Disease Severity

**DOI:** 10.3390/genes11121446

**Published:** 2020-12-01

**Authors:** Tanyaporn Pattarabanjird, Corban Cress, Anh Nguyen, Angela Taylor, Stefan Bekiranov, Coleen McNamara

**Affiliations:** 1 Cardiovascular Research Center, University of Virginia, Charlottesville, VA 22908, USA; tp3cv@virginia.edu (T.P.); cc8en@virginia.edu (C.C.); amt6b@virginia.edu (A.T.); 2 Division of Cardiovascular Medicine, Department of Medicine, University of Virginia, Charlottesville, VA 22908, USA; 3 Division of Cardiology, Duke University School of Medicine, Durham, NC 27710, USA; anhtram.nguyen@duke.edu; 4 Department of Biochemistry and Molecular Genetics, University of Virginia, Charlottesville, VA 22908, USA

**Keywords:** machine learning, deep learning, severity score, coronary artery disease, single nucleotide polymorphism, ID3, coronary angiography, CVD risk prediction, clinical decision making

## Abstract

*Background:* Machine learning (ML) has emerged as a powerful approach for predicting outcomes based on patterns and inferences. Improving prediction of severe coronary artery disease (CAD) has the potential for personalizing prevention and treatment strategies and for identifying individuals that may benefit from cardiac catheterization. We developed a novel ML approach combining traditional cardiac risk factors (CRF) with a single nucleotide polymorphism (SNP) in a gene associated with human CAD (*ID3* rs11574) to enhance prediction of CAD severity; *Methods:* ML models incorporating CRF along with *ID3* genotype at rs11574 were evaluated. The most predictive model, a deep neural network, was used to classify patients into high (>32) and low level (≤32) Gensini severity score. This model was trained on 325 and validated on 82 patients. Prediction performance of the model was summarized by a confusion matrix and area under the receiver operating characteristics curve (ROC-AUC); and *Results:* Our neural network predicted severity score with 81% and 87% accuracy for the low and the high groups respectively with an ROC-AUC of 0.84 for 82 patients in the test group. The addition of *ID3* rs11574 to CRF significantly enhanced prediction accuracy from 65% to 81% in the low group, and 72% to 84% in the high group. Age, high-density lipoprotein (HDL), and systolic blood pressure were the top 3 contributors in predicting severity score; *Conclusions:* Our neural network including *ID3* rs11574 improved prediction of CAD severity over use of Framingham score, which may potentially be helpful for clinical decision making in patients at increased risk of complications from coronary angiography.

## 1. Introduction

Machine learning has emerged as a data-driven technique with the potential to play a role in clinical decision support (CDS) [1]. In the field of cardiovascular medicine, traditional prognostic risk assessments, such as the Framingham Risk Score (FRS), have been used to estimate the 10-year risk of developing coronary heart disease based on data from the Framingham Heart Study. The FRS has been widely used clinically for risk prognostication, decision making related to risk reduction approaches [2,3], and even unsuitability for work [4]. However, subjects at risk for coronary artery disease (CAD) and their doctors face additional decision making dilemmas, such as when to proceed to coronary angiography. Unlike FRS, coronary angiography allows for direct visualization of the coronary anatomy and can aid not only in diagnosis and prognosis of CAD, but also whether revascularization is feasible and/or needed [5]. Yet, traditional risk factors such as male sex, older age, insulin-dependent diabetes and dyslipidemia or FRS alone, while providing 10-year risk assessments, may not provide sufficient predictive power to identify those with severe obstructive CAD [6].

Coronary angiography may be needed in subjects with diagnosed CAD who present clinically with syndromes that may reflect unstable angina or acute coronary syndromes [5]. Yet, symptoms consistent with acute coronary syndromes, such as chest pain with minimal exertion or chest pain at rest, may not be related to CAD even in the setting of multiple cardiac risk factors (CRF) and high FRS [7,8]. Moreover, the diagnostic yield for subjects undergoing elective catheterization is not optimal, with only slightly more than 1/3 of patients undergoing coronary angiography having obstructive CAD [9]. Given the cost and potential risks associated with coronary angiography, better strategies for risk stratification for this group of subjects are needed to support clinical decision-making in the use of coronary angiography [4].

Machine learning has the potential to improve the performance of traditional risk prediction scores by exploiting data repositories to agnostically identify novel risk predictors and more complex interactions between them [10,11,12]. Our aim was to develop a machine learning algorithm integrating conventional risk factors (age, sex, smoking status, blood pressure, fasting blood glucose, HbA1c, total cholesterol, triglyceride, high-density lipoprotein (HDL), low-density lipoprotein (LDL), and body mass index (BMI)), with high-sensitivity C-reactive protein (hsCRP) level and the *ID3* SNP at rs11574 to determine if addition of variables related to inflammation and genetics respectively could improve classification of patients into high and low risk categories for CAD severity (Gensini score) compared to the Framingham score.

Family history, a known risk factor for CAD [13], is thought largely to be due to genetic variants that increase susceptibility to other risk factors along with CAD itself [14,15]. Indeed, genome wide association studies (GWAS) have identified at least 27 genetic variants to be associated with cardiovascular disease [16]. However, heterogeneity of the CAD phenotypes in many GWAS studies, including subject recall of CAD diagnosis or event, population at risk, or various diagnostic or imaging measures do not specifically address the association of the variant with coronary artery atherosclerotic disease severity. A recent study by Zeller et.al. utilized the well-established CAD severity score (Gensini) [17] to determine if known GWAS variants could be associated with CAD severity and found that while the 9p21 variant was associated with CAD severity, others were not [17]. Moreover, gene variants, such as the *ID3* SNP at rs11574, FREM1 SNP at rs10511596, LDLR SNP rs688, and COMT SNP rs4680 [18,19,20,21] associated with direct measures of vessel disease including increased carotid intima-medial thickness (cIMT), coronary artery calcium (CAC) and coronary artery plaque volume as measured by intravascular ultrasound (IVUS) [19,22] have not been identified in GWAS of larger more heterogeneous cohorts. Given our interest in developing a machine learning tool to help predict CAD severity at angiography and our prior work demonstrating that the *ID3* SNP at rs11574 was associated with cIMT, CAC and plaque volume as measured by IVUS in three distinct clinical cohorts, we selected *ID3* SNP at rs11574 as the genetic factor to add to our analysis.

The *ID3* gene encodes the protein Inhibitor of Differentiation 3 (Id3). Id3 is a member of the helix–loop–helix (HLH) transcription factor family and functions as a dominant negative inhibitor of bHLH factor-induced gene regulation. Id3 dimerizes with broadly expressed E-proteins such as E12 and E47 preventing dimerization of these E proteins with each other or other tissue-specific bHLH factors, inhibiting subsequent DNA binding and gene activation by these dimers [23,24,25]. Preclinical studies have clearly shown that atherosclerosis prone mice with global deletion of the ID3 gene have significantly more atherosclerosis at all time points studied in LDLR^−/−^ or Apoe^−/−^ mice, whether Chow or Western diet-fed and by en face or cross-sectional analysis [22,26,27]. Intriguingly, the human ID3 variant at rs11574 that is associated with CAD results in attenuated ability of Id3 to dimerize with E12 and antagonize E12-mediated gene regulation [28]. Yet, whether this human ID3 SNP could improve risk prediction of severe CAD in subjects referred for coronary angiography is unknown. 

## 2. Materials and Methods

### 2.1. Study Design

The cohort includes a total of 481 patients, 32–80 years old presenting to the Cardiac Catheterization laboratory at the University of Virginia (UVA), Charlottesville, for a medically indicated diagnostic cardiac catheterization. Subjects were excluded if they had any of the following: Any acute illness, type 1 diabetes, current acute coronary syndrome (ACS), autoimmune disease, or on immunosuppressive therapy, prior organ transplantation, anemia, pregnancy, and HIV infection. Following cardiac catheterization, the extent of atherosclerotic disease was quantified by using quantitative coronary angiography (QCA) and Gensini score. Traditional risk factors were also assessed through demographic status, physical and physiological measurement, and laboratory values. A detailed description of how Gensini score and each risk factor was quantified is presented below. 

In this study, the main factors included in the development of machine learning models are age, sex, smoking status, BMI, systolic blood pressure, HbA1c, glucose, total cholesterol, HDL, LDL, triglyceride, hsCRP, and Id3 SNP as inputs and Gensini score as an output. 74 patients were excluded from the study after performing outlier filtering described below. High and low Gensini score were defined by first ranking the Gensini score of every patient, and the score ≤ 75 percentile (≤32) was considered low and the score > 75 percentile (>32) was considered high. The remaining 407 patients were randomly categorized into a training set (*n* = 325) and a test set (*n* = 82) to be used in machine learning model development and assessment. 

### 2.2. Assessment of Traditional Risk Factors

Demographic: Demographic description includes age, sex, and medical/social history (including past medical and smoking status)

Physical: Physical description includes height (cm), weight (kg), body mass index as a marker of adiposity (weight (kg)/height^2^ (m^2^)).

Physiological: Physiologic description includes resting blood pressure.

Laboratory Values: All laboratory measures were performed in the CLIA approved clinical laboratory at the University of Virginia. Lipid values included total cholesterol, low-density lipoprotein cholesterol (LDL–c), high-density lipoprotein cholesterol (HDL–c), triglycerides, non-HDL–c cholesterol, hemoglobin A1c (HbA1c), glucose, and high-sensitivity C-reactive protein (hsCRP). 

### 2.3. Genotyping for Id3 Polymorphism

DNA was extracted from whole blood using a Qiagen AutoPure LS (Large Sample Nucleic Acid Purification) automated system using Puregene (Qiagen) chemistry. Using Taqman technology, DNA was genotyped for Id3 polymorphism at SNP rs11574. All the procedures for Id3 genotyping were performed at the UVA Biorepository and Tissue Research Facility (BTRF).

### 2.4. Quantitative Coronary Angiography (QCA)

Patients underwent standard cardiac catheterization with two orthogonal views of the right coronary artery and four of the left coronary artery according to accepted standards. QCA was performed using automatic edge detection at an end diastolic frame. For each lesion, the frame was selected based on demonstration of the most severe stenosis with minimal foreshortening and branch overlap. Computer software was used to calculate the minimum lumen diameter, reference diameter, percent diameter stenosis, and stenosis length. Analysis was performed by blinded, experienced investigators. The Gensini score was used to assign a score of disease burden to each patient. Briefly, each artery segment is assigned a score of 0–32 based on the percent stenosis. The severity score for each segment was multiplied by 0.5–5, depending on the location of the stenosis. Scores for all segments were then added together to give a final score of angiographic disease burden [29]. Score adjustment for collateral was not performed for this study.

### 2.5. Logistic Regression

Multivariate logistic regression was used to predict low and high risk Gensini score based on Framingham risk score and Id3 SNP. The fitted logistic model was determined to be: Gensini low or high=0.0235(FRS)−0.921(Id3 GG)−0.96(Id3 AG)+1.05 and the *p*-values of FRS, Id3 GG, Id3 AG, and Id3 AA are 0.105, 0.262, 0.249, and 0.197 respectively. 

### 2.6. Outlier Filtering

Outliers were detected using Tukey’s method. All the input features (except smoking status, sex, and Id3 SNP) and severity score output were used to calculate IQR (IQR = quartile 3 − quartile 1) for each feature. Outliers were identified by the values less than (quartile 1 − 1.5 × IQR) and more than (quartile 3 + 1.5 × IQR). When the data were visualized by box-and-whisker plots, the outliers beyond the mentioned range were depicted by individual points beyond the whisker regions (Supplementary Figure S1).

### 2.7. Data Normalization and Transformation

All the continuous input features (age, total cholesterol, triglyceride, HDL, systolic blood pressure, HbA1c and glucose) were normalized to a scale of 0–1 in order to minimize the large value effect. Continuous features were then transformed by using the Yeo-Johnson power transformation in order to stabilize variance and reduce skewness of the dataset (Supplementary Figures S1 and S2). Categorical input features (sex, Id3SNP) were transformed by using OneHotEncoder (Supplementary Figure S1).

### 2.8. Feature Selection

Feature selection algorithms were used to select the 9 most important features out of the total 13 input (Supplementary Figure S1). The 3 algorithms used for feature selection includes Spearman correlation between each feature and the Gensini score, a recursive feature elimination wrapper on an XGboost regressor and logistic regression, which identify the most predictive features associated with training and testing boosted decision trees and logistic regression models, respectively. The results from these 3 algorithms were overlapped to obtain the 9 most important features (Supplementary Figure S3). 

### 2.9. Algorithm Development

Various machine learning classification models, including feedforward neural network, deep neural network, Randomforest, Extratree, Adaboost, GradientGoosting, XGBoosting, and support vector machine were applied on the 9 most important features (age, sex, total cholesterol, triglyceride, HDL, systolic blood pressure, HbA1c, glucose, hsCRP and Id3SNP) to classify patients into high and low risk severity score (Supplementary Figures S1 and S4). Each type of machine learning model was optimized on the training set and evaluated on the testing set to identify the most accurate machine learning model. 

### 2.10. Model Analysis

Performance of the models was evaluated by deriving a confusion matrix and area under the curve (AUC) of the receiver operating characteristics (ROC) for each model on the 82 patients of the testing set (Supplementary Figure S4).

## 3. Results

**Figure 1:**
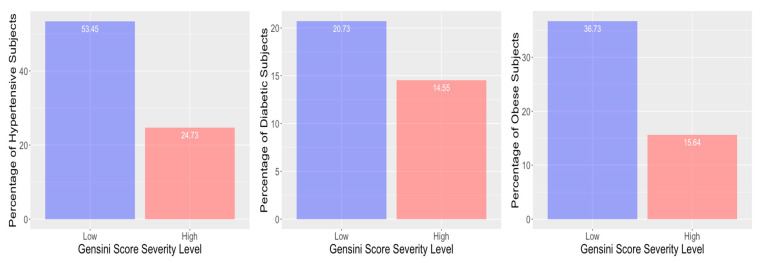
Percentages of subjects with hypertension, diabetes, and obesity in Gensini score low and high groups. Subjects presented at the cardiac catherization clinic were observed to have high prevalence of hypertension (78.18%, with 53.45% in low and 24.73% in high Gensini score severity level), diabetes (35.28 with 20.73% in low and 14.55% in high Gensini score severity level) and obesity (52.37%, with 36.73% in low and 15.64% in high Gensini score severity level).

Consistent with clinical approaches of using pre-test probabilities based on CRF with clinical presentation to make decisions about need for coronary angiography, analysis of the 407 subjects with Gensini scoring for CAD severity in our analysis revealed a high prevalence of patients with hypertension (78.18%), diabetes (35.28) and obesity (52.37%). Surprisingly, there was a higher percentage of subjects with hypertension, diabetes and obesity in the low Gensini score group (Figure 1). Therefore, as hypertension, diabetes, and obesity did not have discriminatory power for severe CAD in our cohort, we excluded these factors as input predictors. 

We divided the 407 subjects into a training set consisting of 325 patients and a testing set consists of 82 patients chosen by random sampling after exclusion of outlier subjects (model performance including outliers is shown in Supplementary Figure S2). There was no significant difference in the input predictors between training and testing sets (Table 1). 

There was a trend toward a lower percentage of female and a higher percentage of subjects with low Gensini scores in the training set (severity score distribution in training and testing sets are visualized in Supplementary Figure S5). However, the difference in these proportions should not affect model training and performance evaluation significantly given that the differences in percentages were not statistically significant; a sufficient number of training and test samples were available; and, model performance was evaluated by measures that account for both false positives and negatives including the ROC AUC and confusion matrix. 

### 3.1. Principal Component Analysis and Correlation Analysis of the Input Features

**Figure 2:**
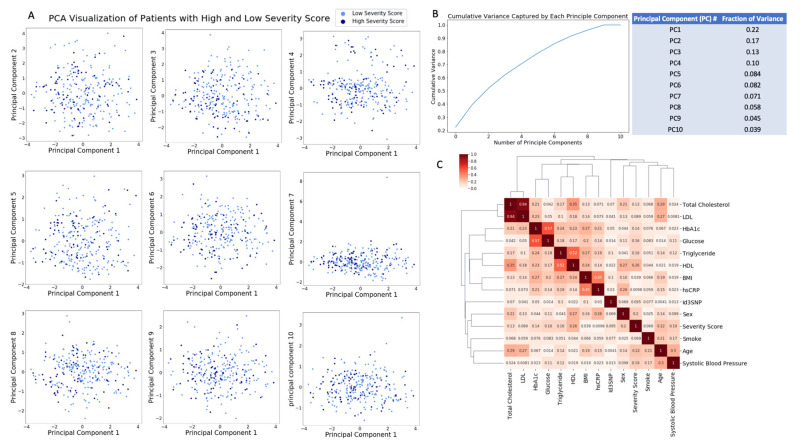
PCA and correlation matrix of patients with high and low severity score generated using conventional risk factors and Id3 SNP. PCA plots between PC1 and PC2-PC10 (**A**) and low fraction of variance captured by each principal component (PC) (**B**) suggested that patients with high and low severity score cannot be differentiated by using PCA based on their conventional risk factors and Id3 SNPs. Correlation matrix also demonstrates low-intermediate correlation between severity score and conventional risk factors and Id3 SNP (**C**). Both PCA and correlation matrix analysis indicates a challenge in developing a model/strategy to classify patients into high and low risk severity score.

Principal component analysis (PCA) is commonly used to visualize separation of samples according to treatment, cell state, genotype, etc. in a two-dimensional plot based on high dimensional data. In this sense, it is known as a dimensionality reduction technique which allows features to be extracted using the main axes of the plot (i.e., principal components). Applying PCA on all the input features can help facilitate discovery of a combination of input features that enables classification of high and low severity score patients. Visualization of the result from applying PCA to all the risk variables and Id3 SNP genotype (Figure 2A) indicates that 10 principal component (PC) were required to capture the variance of the dataset and none of the PCs can separate patients into high and low severity score based on their conventional risk factors and Id3SNP genotype (Figure 2B). Correlation analysis was performed to characterize the association between each input feature and Gensini score using the Spearman correlation coefficient. Spearman correlation was chosen due to a lack of normality in most of the input variables and the output of the patient dataset. Correlation analysis also indicates low to intermediate correlation between input features and the severity score output with the 3 highest correlations being 0.26 with HDL, 0.22 with age, and 0.2 with sex (Figure 2C). PCA and correlation analysis demonstrate the challenge of using conventional risk factors and Id3 SNP to classify patients into low and high Gensini score severity level.

### 3.2. Use of Framingham Score to Classify Patients into High- and Low-Risk Severity Score

**Figure 3:**
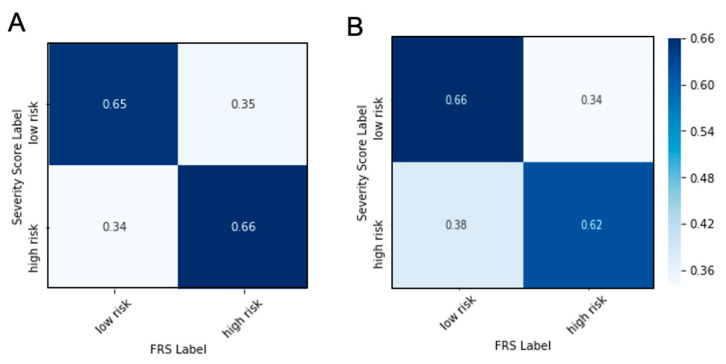
Performance of high and low risk FRS in estimating high and low risk severity score. Framingham Risk Score (FRS) can accurately predict low risk group with 65% accuracy and high risk group with 66% accuracy (**A**). With addition of ID3 SNP to FRS, the model predicts low risk group with 66% accuracy and high risk group with 62% accuracy (**B**).

As mentioned earlier FRS is a gold standard used in clinical practice to mainly aid decision making related to risk reduction approaches. Gensini severity score, on the other hand, is used to evaluate extent of CAD. A patient with an FRS > 10 is considered at high risk of developing cardiovascular disease, and a patient with a Gensini severity score > 32 is considered to have high CAD severity suggesting a possible need for revascularization. When FRS was tested to evaluate Gensini Severity Score, high FRS predicts high Gensini score with 66% accuracy and low FRS predicts low Gensini score with 65% accuracy (Figure 3A). The additive value of Id3 SNP to FRS in predicting Gensini score was evaluated by constructing a multivariate logistic regression model. The performance of this logistic model evaluated by likelihood ratio test (*p* = 0.267), Akaike information criterion (AIC = 261.59), and Nagelkerke pseudo R^2^ (pseudo R^2^ = 0.027) indicates a lack of good model fit. This caveat potentially results from an insufficiency of FRS and Id3 SNP alone to explain the data’s variance as well as a lack of model complexity. Assessing the input variables in the logistic regression analysis, we found that none of the Id3 allele variants at rs11574 was significant for predicting low and high risk Gensini scored (*p* = 0.262 for GG, *p* = 0.249 for AG, *p* = 0.197 for AA), and FRS was only trending significant (*p* = 0.107). Furthermore, we used positive predictive value (PPV) and negative predictive value (NPV) to evaluate all the models in this study, which are accuracy measures associated with the fraction of correct calls in each of the two classes, low and high Gensini score. PPV is used to measure the accuracy of the model in predicting patients with high Gensini score severity to be in the high Gensini score group. Similarly, NPV measures the accuracy of the model in predicting patients with low Gensini score severity to be in low Gensini score group. When confusion matrices pre- and post-addition of Id3 genotype at rs11574 were compared, no significant change in either PPV or NPV was observed as PPV increased from 65% to 66% while NPV decreased from 66% to 62% (Figure 3B). This indicates the necessity of developing new models that can specifically predict risk of having severe coronary artery disease as measured by coronary angiography. 

### 3.3. Structure and Performance of Best Performing Model: Sequential Neural Network

**Figure 4:**
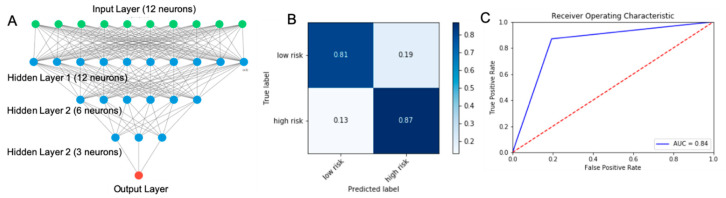
Structure and performance of our best machine learning based model in predicting high and low risk severity score evaluated by confusion matrix and AUC of ROC. The best machine learning model with the highest performance is a deep neural network model with 12 neurons in the input layer including age, total cholesterol, triglyceride, low-density lipoprotein (LDL), high-density lipoprotein (HDL), systolic blood pressure, HbA1c, glucose, sex and Id3SNP after all these inputs were normalized and transformed. The model contained 12 and 6 neurons in the next sequential layers and an output layer (**A**). The model performance was evaluated on 82 patients in the testing set. With our best model, the low risk group was predicted NPV of 81% and high risk group with PPV of 87% (**B**). ROC plot showing the performance of the model (blue line) with an AUC of 0.84 and random (dashed red line) for comparison (**C**).

Various machine learning algorithms were trained and tested using 9 selected input features (age, sex, total cholesterol, triglyceride, HDL, systolic blood pressure, HbA1c, glucose, and Id3 SNP variants) to predict low and high Gensini score severity. The performance of the model was evaluated by PPV, NPV, AUC, of ROC, and confusion matrix as metrics. The best algorithm is a sequential neural network, which is a hierarchical organization of artificial neurons passing messages and forming complex interactions with other artificial neurons based on the received inputs. The input data is utilized by the first layer of neurons, which then parse the outputs to the consecutive layers of neurons, and finally compute the final predictive outcomes. Architecture of our model consists of 12 neurons in the first layer, 6 neurons in the second layer, 3 neurons in the third layer and an output layer (Figure 4A and Supplemental Figure S4). Our model can predict high Gensini severity score (>32) with PPV of 87% and low Gensini severity score (≤32) with NPV of 81% (Figure 4B) with an ROC AUC of 0.84 (Figure 4C).

### 3.4. ID3 Allele Variants at rs11574 Enhances Prediction Accuracy of the Neural Network Model

**Figure 5:**
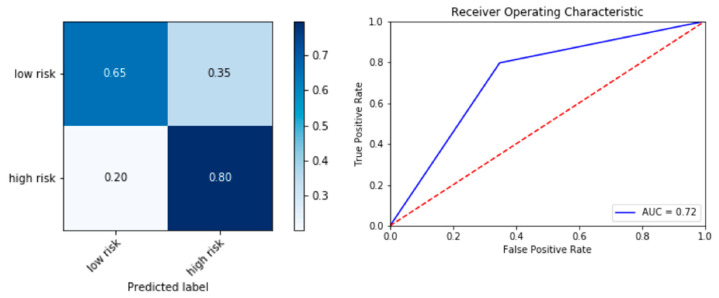
Elimination of Id3 SNP as input variable reduces model performance. Performance of the model drops when Id3 SNP was removed. Positive predictive value (PPV) of predicting high risk group drops from 87% to 80% and NPV of predicting low risk group drops from 81% to 65%. AUC of ROC also drops from 0.84 to 0.72.

Id3 SNP at rs11574 has been shown to be associated with subclinical CAD. To understand the importance of Id3 SNP variants on the performance of the neural network model, the Id3 SNP input feature was omitted and the same optimized procedure (Supplemental Figure S1) was implemented to evaluate the performance of the model. The PPV was reduced from 87% to 80% in the high Gensini severity score group and the NPV 81% to 65% in the low Gensini severity score group, while AUC of ROC was reduced from 0.84 to 0.72 (Figure 5). Net reclassification improvement (NRI) score was also calculated to compare the model performance before and after addition of Id3 SNP [30]. Categorical NRI was calculated to be 0.0975 (*p*-value = 0.0872), suggesting a trending significance of Id3 SNP addition to the neural network model.

### 3.5. Contribution of Each Input Feature on Performance of the Neural Network Model

**Figure 6:**
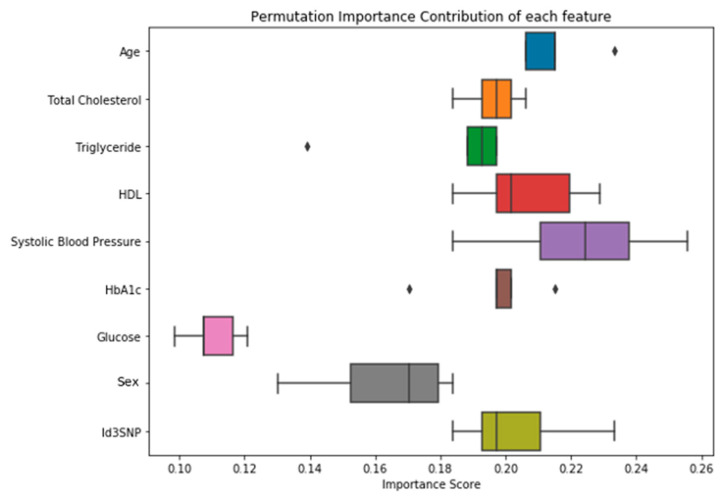
Importance score of each input feature demonstrated with different color boxplot (with square indicating outliers) on our best machine learning model. Importance score, defined as cross-validation score for model with actual values minus that using permuted values, for each of the 9 most important features where values were permuted 5 times for each feature. Age, total cholesterol, triglyceride, HDL, systolic blood pressure, HbA1c, glucose, sex, and Id3SNP are the 9 most important features out of 13 features chosen as inputs of our model input features. These 9 inputs contribute at different levels in enhancing precision and accuracy of the model with systolic blood pressure, HDL and age being the top 3 most important inputs.

Permutation feature importance was used to measure the increase in the prediction error of the model after each features’ values were randomly permuted. The purpose of permuting values of each feature is to break the relationship between the feature (e.g., HDL) and the outcome (i.e., high or low Gensini score group). After permutating the value of all the input features one at a time and then entering them into the neural network model, change in cross validation score (defined as importance score) was calculated. The 7 most important input features with the highest changes in median cross validation scores are, in order of importance, systolic blood pressure, age, HDL, HbA1c, total cholesterol, Id3 SNP, and triglyceride, all of which had median scores above 0.198 (Figure 5). Notably, systolic blood pressure importance scores were not significantly different (*p*-value = 0.138) from the next most important variable, age, while fasting blood glucose and sex importance scores were significantly lower (*p*-value = 0.0431; Wilcoxon test between sex and triglyceride) than that of the other variables (Figure 6). 

## 4. Discussion

Severe atherosclerosis can develop decades prior to clinical manifestations. Given that aggressive risk factor modification can reduce the risk of adverse cardiac events, identification of asymptomatic individuals with advanced atherosclerosis is of clear significance [31]. On the other hand, chest pain, a concerning symptom that frequently results in advanced cardiac imaging or hospitalization, may not be due to advanced atherosclerosis. As such, development of more predictive algorithms based on clinical information and laboratory measures to identify patients with severe coronary artery disease may assist in clinical decision making that could improve prevention of adverse cardiac events and avoid more costly and higher-risk tests in low-risk patients. 

We selected and optimized a top-performing sequential neural network model from many other machine learning models to predict CAD severity as defined by high (>32) and low (<32) Gensini severity score. Commonly measured clinical variables and SNP in the ID3 gene at rs11574 were used as inputs. We specifically chose the ID3 SNP at rs11574 as it has not been identified by GWAS as associated with CV events, but has been shown to be associated with severity of CAD as measured by IVUS. Predictions of high and low Gensini severity score from this neural network model may hold promise for (1) patients at high risk of complications with coronary angiography being considered for this procedure, and (2) patients presenting with non-specific chest pain at the emergency room to help inform the likelihood that it is due to CAD. This work also demonstrates that the SNP in the *ID3* gene at rs11574 may be an important modifier for risk of severe CAD as inclusion of the *ID3* SNP genotype as an input feature enhances accuracy of the neural network model significantly. Notably, all the clinical features and laboratory values used in our model, and hsCRP level can easily be obtained at point of care. A GeneDrive SNP variant assay has recently been developed for point of care application [32], which could allow for the *ID3* SNP variant or other gene variants, to be easily incorporated into an app-based calculation of CAD severity risk. This study is a proof of concept that machine learning models may have utility at point of care to evaluate patients with CAD or at risk for CAD based on high risk coronary anatomy as assessed by Gensini score.

The present study also highlights a role for testing CAD associated SNPs not identified in GWAS in machine learning models to potentially enhance predictive accuracy. While GWAS has identified > 27 gene loci associated with CAD and MI [14,33], they only explain 15–20% of CAD heritability [16]. Interestingly, the *ID3* SNP at rs11574 has not been identified as a risk SNP in GWAS. Yet, studies testing for the association of this SNP with direct measures of atherosclerosis in humans have shown a stepwise increase in cIMT in subjects from the Diabetes Heart Study (DHS) with one or two alleles containing the *ID3* variant at rs11574 [22]. Consistent with these findings, the *ID3* SNP at rs11574 was similarly associated with the amount of coronary artery calcium (CAC) in participants in the multiethnic study of atherosclerosis (MESA) and plaque burden as measured by IVUS in the CAVA cohort [19]. Notably, biochemical analysis of the Id3 protein encoded by the allelic variant associated with atherosclerosis revealed an attenuated ability of the protein to perform its functions as a dominant negative regulator of gene transcription. Murine studies have provided clear evidence that loss of Id3 promotes atherosclerosis in both Apoe^−/−^ [22,27]. and LDLR^−/−^ mice [26].

Our study has limitations. First while the Gensini score is a widely used scoring system for coronary angiography to evaluate severity of CAD, it is not as commonly used to evaluate difficulty of revascularization (e.g., percutaneous coronary intervention (PCI) and coronary artery bypass grafting (CABG)) as Syntax score. Yet, studies have demonstrated a significant correlation between Gensini severity score and Syntax score with Gensini score > 50.5 predicting need for revascularization via CABG with 77% sensitivity [34]. An important advantage of using the Gensini score over others (e.g., Syntax score) is that it includes early alterations of atherosclerotic disease and mild stenosis. Taken together, these results suggest that patients with Gensini Severity Score of >32 have a higher likelihood to potentially need revascularization. Second, we did not evaluate family history (FH) of CVD in our analysis. FH has been long been known as a risk factor for CAD. Although accuracy of patient reports of their family members’ disease status can be moderately low [35], it might still meaningfully improve prediction of Gensini CAD severity score. Our study was limited by the absence of this factor’s collection in our dataset. This factor should be considered in future development of machine learning models. Third, as shown in Supplementary Figure S2, our machine learning model was only applied to individuals with clinical parameters (age, total cholesterol, triglyceride, HDL, systolic blood pressure, glucose, and HbA1c) in the range between a lower and upper bound. According to the fact that hypercholesterolemia is a known co-morbidity of CAD, there is a possibility that individuals who will be tested by this machine learning model might have total cholesterol (>254 mg/dL) and triglyceride (>278 mg/dL) that fall beyond the range in which the model was optimized. These individuals will have to be excluded from application of this model. In addition, this study was done in a small cohort of 481 patients. This small cohort precluded our ability to divide the subjects into three groups (high, intermediate and low). Instead, we defined two groups by first ranking the Gensini score of every patient, and utilizing those above the 75th percentile as high and those below as low. It will clearly be important to validate our machine learning model in a larger cohort of individuals with CAD severity assessed by angiography allowing the assessment of high, low and intermediate groups. Yet, taken together results provide proof of concept that readily-obtainable clinical data in conjunction with genetic SNPs associated with severity of CAD (even if subclinical) may improve prediction of severe CAD as measured by coronary angiography.

## Figures and Tables

**Table 1:** Patients characteristics of training and testing set.

Variable	Training (*n* = 325)	Testing (*n* = 82)	*p*-Value
Age (mean ± SD)	63.7 ± 9.7	61.0 ± 10.3	0.5466
Sex (*n*%, female)	38.5%	53.5%	0.2883
BMI (mean ± SD)	31.4 ± 6.1	31.5 ± 6.6	0.9259
Total Cholesterol (mean ± SD)	153.8 ± 33.9	159.39 ± 33.8	0.4585
Triglyceride (mean ± SD)	108.6 ± 57.8	111.9 ± 49.5	0.6333
HDL (mean ± SD)	40.9 ± 11.2	42.7 ± 14.5	0.7685
LDL (mean ± SD)	94.8 ± 29.9	98.1 ± 29.8	0.3115
Glucose (mean ± SD)	118.5 ± 42.6	107.44 ± 27.9	0.4586
HbA1c (mean ± SD)	6.33 ± 1.29	6.06 ± 1.03	0.6407
hsCRP (mean ± SD)	2.99 ± 3.29	3.09 ± 2.73	0.8462
Systolic Blood Pressure (mean ± SD)	136.0 ± 18.4	133.04 ± 19.9	0.5972
Current Smoker (% *n*)	14.3%	10.7%	0.3459
Id3 SNPs (% *n*, GG, AG, AA)	60.3%, 35.1%, 4.5%	61.8%, 32.7%, 5.5%	0.7756
Severity Score (mean ± SD)	21.5 ± 20.2	16.6 ± 16.4	0.2562
Proportion of Patients with Low Risk Severity Score	75.0%	60.7%	0.2742

## Supplementary Materials

The following are available online at https://www.mdpi.com/2073-4425/11/12/1446/s1, Figure S1: Machine Learning Pipeline. Figure S2: Interquartile Range (IQR) method for outlier filter. Figure S3: Applying Yeo-Johnson power transformation to stabilize variance, minimize skewness, and increase normality of each input feature. Figure S4: Feature selection algorithms used to select important features to develop our machine learning model. Figure S5: Severity score distribution in both training and testing set. Figure S6: Various machine learning models were tested to select the highest performing machine learning model. Figure S7: Sequential neural network algorithm. Figure S8: Mean square error loss function, Adam optimizer, L2 regularization with λ = 0.01 and implementation of early stop by monitoring testing accuracy yield the highest model performance.
