# Supplementary material for: A Machine Learning Model Utilizing a Novel SNP Shows Enhanced Prediction of Coronary Artery Disease Severity
Source: Genes (Basel). 2020 Dec 1;11(12):1446. doi: 10.3390/genes11121446 (PMC7760379; doi:10.3390/genes11121446)
Supplement: Supplementary file 1 [file genes-11-01446-s001.pdf]

Supplementary Material

1. Machine learning pipeline

A comprehensive machine learning pipeline is shown in Figure S 1 and described in more details below.
Step 1 Assigning task: we set a classifying task for this machine learning model by assigning potential inputs and outputs. The input parameters consist of age, sex, smoke, BMI, systolic blood pressure, HbA1c, glucose, HDL, LDL, smoking status, total cholesterol, triglyceride, hsCRP and Id3SNP. The outputs are Genisi severity score of > 32 (> 75 percentile) and Gensini severity score of ≤ 32 (≤ 75 percentile).
Step 2 outlier filters: outliers were removed in order to prevent the model from overfitting the outliers.
Step 3 data transformation: all the data were normalized and transformed to reduce large number effect and introduce normality to each input feature.
Step 4 feature selection: important features were selected by using various feature selection algorithms in order to avoid model overfitting and reduce computational time.
Step 5 train-test split: total data were randomly divided into 2 groups, training (80%) and testing (20%) sets. Preserving 20% of total data as a testing set is essential for accurately evaluating the model performance since the model will never see the testing set data.
Step 6 model selection and optimization: Major machine learning models commonly used for classification tasks include support vector machine (SVM), tree/ensemble-based models, and neural network models. All types of these models were tested to choose the best performing model. For each model, model parameters were optimized and optimization was executed by using 10-fold cross-validation.

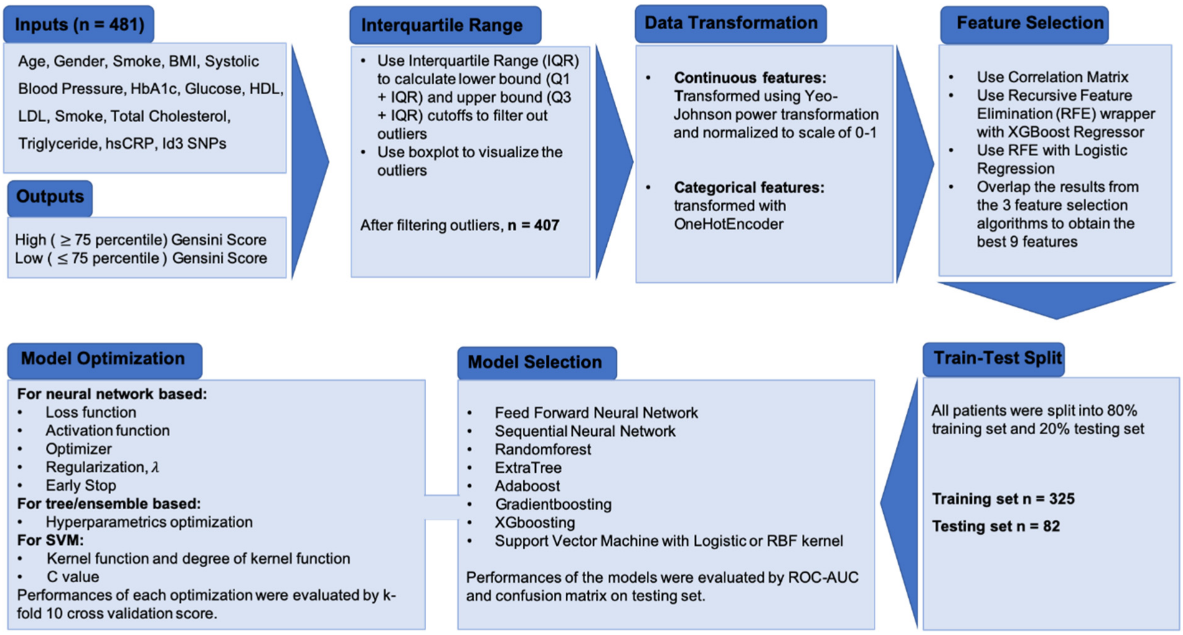

Figure S1: Machine Learning Pipeline. Pipeline of machine learning model development includes 3 main steps: 1) defining inputs and outputs 2) data pre-processing (feature selection, data transformation), and 3) model optimization and model selection.

2. Interquartile range outlier filter

Box-whisker plot is a standard plot commonly used to visualize data distribution and highlight

outliers. Outliers in box-whisker plot are calculated by using interquartile range with the lower bound of (Q1 – 1.5xIQR) and upper bound of (Q3 + 1.5xIQR) [1]. Box-whisker plots of our data and also lower and upper bound for outlier filtering are shown in Figure S2. In order to evaluate the effect of the outliers on the machine learning model performance, the optimized sequential neural network was also applied to the total number of subjects. The result evaluated by confusion matrix and ROC-AUC indicates that the prediction highly skewed towards low risk prediction and prediction accuracy was compromised (Figure S2).

Features	Lower bound (Q1 - 1.5 x IQR)	Upper bound (Q3 + 1.5 x IQR)
Age	33.5	93.5
Total Cholesterol	54	254
Triglyceride	0* (-58)	278
HDL	9.5	69.5
Systolic Blood Pressure	86	182
Glucose	53.5	161.5
HbA1c	4.25	7.85
Severity Score	0* (-43)	85

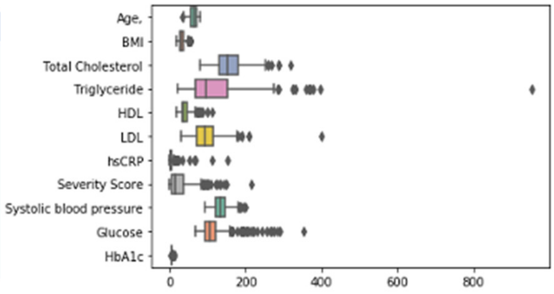

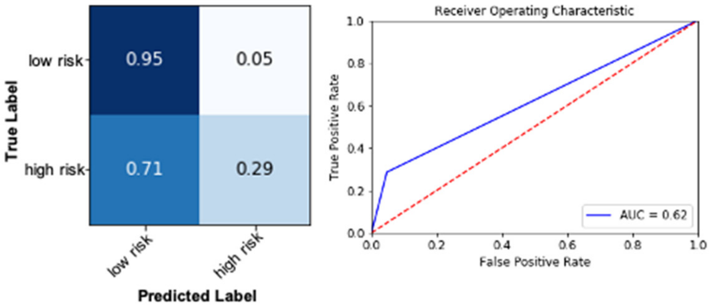

Supplementary Figure 2: Interquartile Range (IQR) method for outlier filter and model performance with inclusion of outliers. Quartile 1 (Q1), Quartile 3 (Q3), and IQR (Q3-Q1) were calculated, and lower bound cutoff (Q1– 1.5 x IQR) and upper bound cutoff (Q3 + 1.5 x IQR) for each feature are shown in the table (left). The data for each feature were visualized by box-whisker plots (right). Optimized sequential neural network model was also applied to overall subjects prior to exclusion of outliers and evaluated by confusion matrix and ROC-AUC (below). * indicates the lowest measurable values.

3. Data transformation and normalization

Continuous input data were transformed by using Yeo-Johnson power transformation [2] with maximum likelihood estimator, λ [2]. Yeo-Johnson transformation is in the same family as Box-Cox family but does not require input values to be strictly positive. This family of power transformation is commonly used to transform non-normal data into a more normally distributed data. This transformation is essential since normality is either required for or improves performance of various machine learning approaches. Increased data normality after transformation and associated λ that maximizes likelihood estimation for each feature are shown in Figure S3. These transformed data were then normalized by using a robust scaling approach (equation shown below) which avoids scaling data by the maximum value which tends to be unrobust. Categorical input data were transformed with OneHotEncoder [3]. OneHotEncoder works by splitting categorical variables (e.g., sex = male or female) into multiple logical (true/false) variables (e.g., separate male and female categories) [3] which take the values 1 (true) or 0 (false). For example, in the case of a female, female = 1 and male = 0).

Normalization: $N(x_i) = \frac{2}{1+e^{-2x_i/u}} - 1$; where $u = 95th\ percentile\ of\ \forall x_i$

Yeo-Johnson transformation:

$$f(x) = \begin{cases} ((x+1)^{\lambda}-1)/\lambda; \text{ if } \lambda \neq 0, x \geq 0 \\ \log(x+1); \text{ if } \lambda = 0, x \geq 0 \\ -[(-x+1)^{2-\lambda}-1]/(2-\lambda); \text{ if } \lambda \neq 2, x < 0 \\ -\log(-x+1); \text{ if } \lambda = 2, x < 0 \end{cases}$$

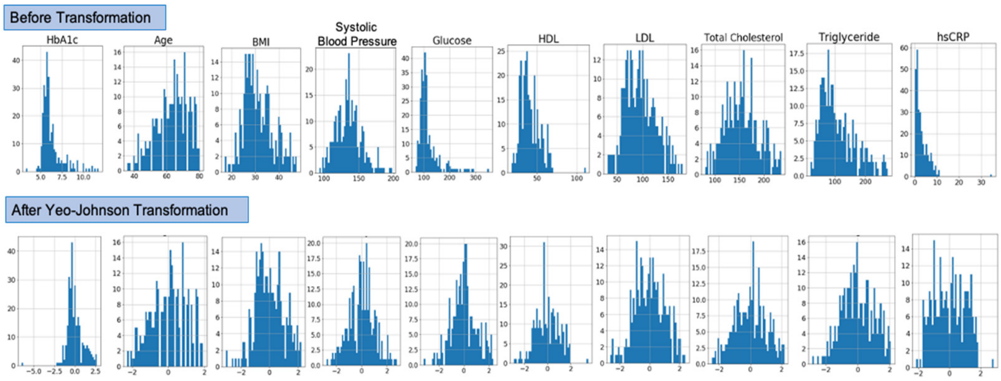

Features	λ value for Yeo-Johnson power transformation
Age	2.11
BMI	-0.083
Total Cholesterol	0.547
Triglyceride	0.0405
HDL	-0.123
LDL	0.499
hsCRP	-0.297
Systolic Blood Pressure	-0.215
HbA1c	-2.01
Glucose	-1.82

Figure S3: Applying Yeo-Johnson power transformation to stabilize variance, minimize skewness and increase normality of each input feature. Histogram plots (left) of the data before (above) and after (below) indicated an increase in normality of the data after transformation. Table showing each λ values for each feature (right) used for Yeo-Johnson transformation calculated using maximum likelihood estimation.

4. Feature Selection

We used the Spearman correlation between Gensini severity score and each feature to perform feature selection together with feature elimination approaches. Features were selected if they passed at least two of the three criteria. For the Spearman criteria, features passed if their correlation coefficient > 0.08. The two feature elimination approaches were recursive feature elimination (RFE) [4] using an XGBoost regressor model [6] and a logistic regression model where the features are selected based on a cumulative local maximum in the changes of a cross validation score. Briefly, Spearman correlation between two variables is a rank-based approach to quantitatively assess their association. Perfect correlation, anti-correlation and no correlation yield Spearman correlation coefficients of +1, -1 and 0, respectively. RFE fits a specified model and removes a specified number of the weakest features. These weakest features are identified through recursively eliminating features per iteration and features are then ranked by the specified model's accuracy score [3]. Therefore, RFE on XGBoost regressor and on logistic regression indicate XGBoost and logistic regression fitted models [5]. Applying the Spearman correlation feature selection criteria detailed above yielded the following 9 features: age, total cholesterol, triglyceride, HDL, systolic blood pressure, HbA1c, glucose, sex and Id3 SNP (Figure S4). By using RFE on XGBoost regressor, the optimal number of features is 10 with the same 9 features indicated by Spearman correlation and hsCRP (Figure S4). By using RFE on logistic regression, the optimal number of features is 9: age, total cholesterol, triglyceride, HDL, LDL, HbA1c, sex, smoking status and Id3SNP (Figure S4). The overlap from all three approaches yielded 7 features: age, total cholesterol, HDL, HbA1c, sex and Id3 SNP (Figure S4). Applying our overall criteria of features passing at least two criteria, we arrived at the following 9 input features to further assess various machine learning models: age, total cholesterol, triglyceride, HDL, systolic blood pressure, HbA1c, glucose, sex and Id3 SNP.

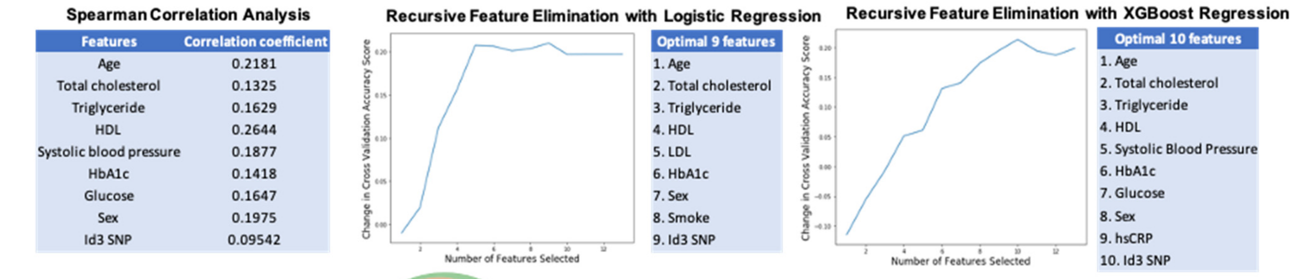

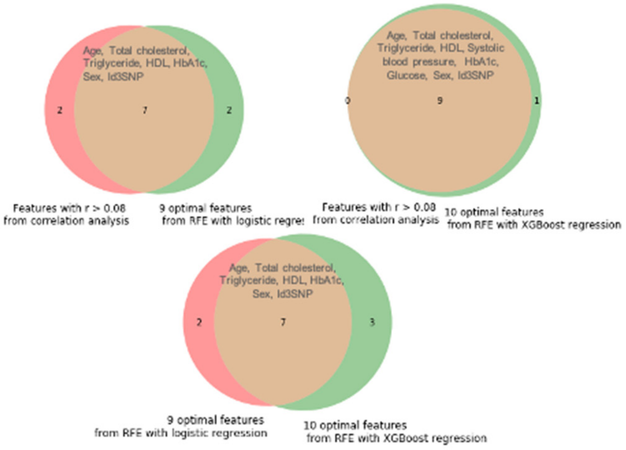

Figure S4: Feature selection algorithms used to select important features to develop our machine learning model. Spearman correlation analysis, recursive feature elimination (RFE) with logistic regression, and RFE with XGBoost regression were 3 algorithms used to select the most important features for machine learning model development. This feature selection process helps with reduction of the variance and overfitting of the model. By overlapping results from the mentioned algorithms, age, total cholesterol, HDL, HbA1c, Sex and Id3 SNP are the most 7 important features. Systolic blood pressure and glucose were added to our model due to the relative strength of their decent correlation coefficient.

5. Splitting training and testing sets

All patients were randomly split into 2 groups with 80% of all patients (n = 325) to be in a training set and 20% of all patients (n = 82 patients) to be in a testing sets by using a hold-out approach. The testing set is only used for evaluating the performance of the model, but not optimizing the model. Patients characteristics were not significantly different between training and testing sets as described in Table 1. However, proportion of patient with low risk severity score was higher in the training set despite higher average severity score. To further understand this phenomenon, distribution of severity score in training

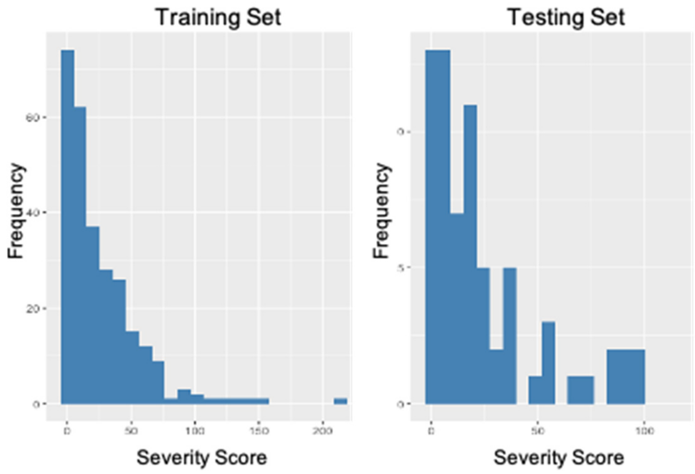

Figure S5: Severity score distribution in both training and testing set.

and testing set was visualized through histogram plots in Figure S5. Severity score distribution demonstrated that training set contains some subjects with severity score higher than 100 while testing set contains none. This might be a potential cause explaining the phenomenon.

6. Model selection and optimization

We chose SVM models [6], tree/ensemble-based models (Randomforest [7], ExtraTree [8], Adaboost [9], GradientBoosting[11], XGBoost [5]), and neural network-based models (feedforward neural network and sequential neural network)[35] for our high and low Gensini Severity Score classification due to their generally good performance on classification tasks. Each type of model consists of different type of parameters required for optimization. The optimization was done on the training set using 10 fold-cross validation. Performance of each optimized configuration was evaluated by using the F1 cross validation score (F1 = $2x\frac{precision\ x\ recall}{precision + recall}, precision = \frac{True\ positive}{True\ positive + False\ positive}, recall = \frac{True\ positive}{True\ positive + False\ negative}$).

6.1. Brief description of each model

SVM: an algorithm that creates a line or a hyperplane which separates data into classes. Kernel and soft SVM are additional approaches that allow SVM to solve non-linear problems [6].
Tree/ensemble: a family of algorithms that performs classification from many decision trees. Introduction of bagging and boosting methods has increased prediction performance of this family of algorithm significantly. Bagging is a method to create several subsets of data from training samples and use each subset to build a decision tree. Average prediction of all decision trees will be used to predict the final result. Boosting is a method to fit consecutive decision trees at every step and use the error learned from the prior iteration to penalize input features driving the prediction error during the next round of model building and assessment. Randomforest and ExtraTree are the two algorithms that utilize the bagging method, and Adaboost, GradientBoosting and XGBoosting are the three algorithms that use the boosting method[5,6,9,11].

Neural network: a family of algorithms that iteratively weights contribution of each input feature that feed into non-linear neuron-like elements to find the best set of weights that accurately predict the output. After each input is weighted, summation of all weighted inputs is calculated before being input into non-linear activation functions to predict the output. A feedforward neural network is the simplest neural network with direct connection between input and output layers, while deep neural network contains multiple layers in between the input and output layers [12]. Performance of each machine learning models is shown in Figure S6.

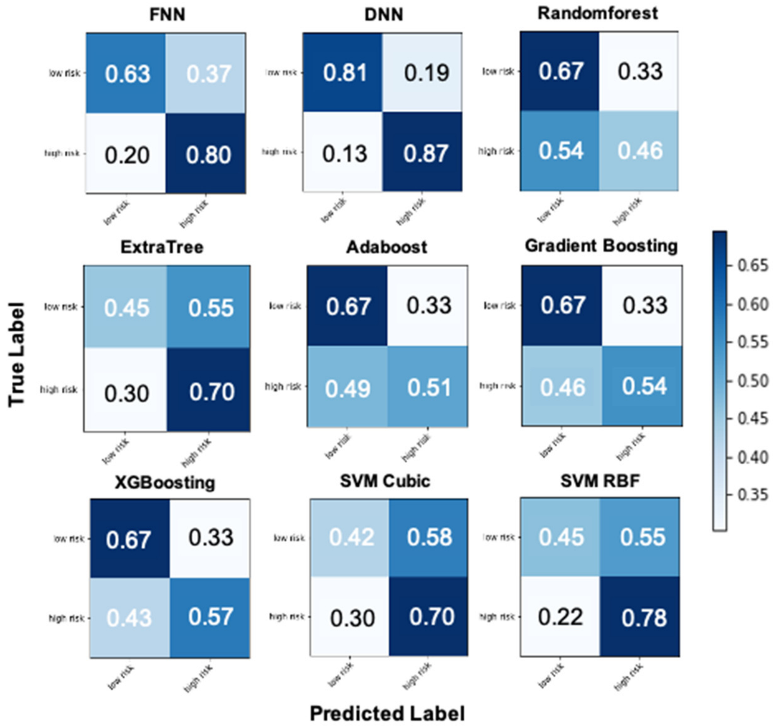

Model	ROC-AUC
Feedforward Neural Network (FNN)	0.71
Deep Neural Network (DNN)	0.84
Randomforest	0.56
ExtraTree	0.58
Adaboost	0.59
Gradient Boosting	0.60
XGBoosting	0.62
SVM with cubic polynomial kernel	0.56
SVM with RBF kernel	0.58

Figure S6: Various machine learning models were tested to select the highest performing machine learning model. The tested models include neural network based (feedforward and sequential), tree and ensemble based (Randomforest, ExtraTree, Adaboost, Gradient Boosting, and XGBoosting), and support vector machine based (cubic polynomial and RBF kernels). Sequential or deep neural network (DNN) was evaluated by confusion matrix and ROC-AUC to be the best model.

6.2. Optimizations

SVM models: we optimized kernel functions (RBF function and polynomial functions), degree of kernel function (degree of 1 to 7), and C value (degree of 1 to 3 for soft SVM penalty).

Tree/ensemble-based models (Randomforest, ExtraTree, Adaboost, GradientBoosting, XGBoost): we optimized number of trees, number of features considered at each split, maximum number of tree splitting, minimum number of samples for each split, and method of selecting samples for training each tree (bootstrap).

Neural network-based models (feedforward, sequential neural network): we optimized types of loss function, types of activation function, types of optimizer, types of regularization and its best λ to minimize the model loss, learning rate, and early stop implementation.

Sequential Neural Network

The Sequential (or deep) neural network (SNN or DNN) was demonstrated to be the best machine learning model with the highest accuracy when fitted with our data. SNN is a type of a fully connected neural network with multiple hidden layers in between an input layer and an output layer. These hidden layers facilitate transformation of information from the input data and detecting relevant inputs by reducing dimensionality while allowing as many inputs as necessary. Detailed algorithm and mathematical representation of the model can be found at Denoyer et al (2014) [12].

Model architecture: Our model consists of a 12-neuron input layer, 12-neuron first hidden layer, 6-neuron second hidden layer, 3-neuron third hidden layer, and an output layer. The 12-neuron input layer receives 7 continuous inputs after transformation and normalization (age, total cholesterol, triglyceride, HDL, systolic blood pressure, HbA1c, and glucose) and 5 categorical inputs after OneHotEncoder transformation (male, female and three Id3 SNP genotypes). In every neural network layer, a weight and bias matrix was applied on the incoming matrix (X for input layer, h1 for 1st hidden layer, h2 for 2nd hidden layer, and h3 for 3rd hidden layer). The resulting matrices after applying weight and bias were passed through activation functions. Neuron-like non-linear elements are implemented by activation functions which require the sum of weighted inputs to exceed a threshold in order to "fire" or output a non-zero value. An activation function is applied to every layer with a rectified linear unit (ReLU) as the activation function of every layer except the output layer (shown in dark blue boxes in Supplementary Figure 6) and a sigmoid as an activation function of the output layer (shown in a green box in Figure S7). Once the input/output data propagate through all the layers to the last sigmoid activation function, the resulting vector $\hat{y}$ is the predicted output vector. Using training set data, this predicted output was compared with the assigned output by calculating the mean square error loss function (E). Backpropagation was used to calculate the gradient of the loss function with respect to the weights of the neural network, thereby arriving at weights that minimize the difference between the model prediction and actual output data. The Adaptive Moment Estimator (Adam) optimizer was the best performing gradient method which we used to minimize model loss or optimize our model [10].

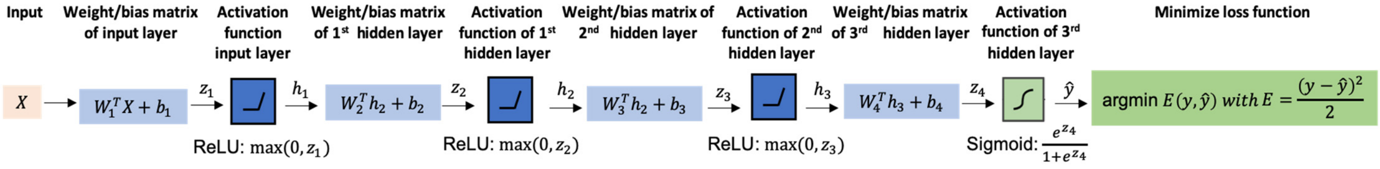

X = *input matrix*
W_i = *L2 regularized weigh matrix*
b_i = *L2 regularized bias vector*
$z_i = W_i^T X + b_i$
h_i = *resulting vector after passing through activation function*
$\hat{y}$ = *predicted output vector*
y = *known output vector*
E = *mean square error loss function*

Figure S7: Sequential neural network algorithm. Input matrix was passed through 4 layers including an input layer and 3 hidden layers. In each layer, L2 regularized weight and bias matrices were applied and the resulting vector was passed through activation functions. ReLU activation functions were used for the input layer and first two hidden layers. A sigmoid activation function was used as a final activation function to output the prediction layer. Backward propagation was applied to calculate the mean square error (MSE) and the MSE was minimized by using the Adam optimizer.

Model fitting: The aim of model fitting is to optimize the predictive power of the model or minimize the model loss. After model optimization, we determine best loss function to be a mean square error loss function with L2 (ridge) regularization model complexity penalty parameter $\lambda = 0.01$ to optimally minimize loss, and Adam as the best optimizer.

Adam optimizer:

$$\theta_{t+1} = \theta_t - \frac{\eta}{\sqrt{\hat{v}_t + \epsilon}}\hat{m}_t; \ \epsilon = 10^{-8} \quad (1)$$

$$\hat{m}_t = \frac{m_t}{1-\beta_1} \text{ and } \hat{v}_t = \frac{v_t}{1-\beta_2} \text{ (for bias correction)}; \beta_1 = 0.9, \ \beta_2 = 0.999 \quad (2)$$

$$m_t = \beta_1 m_{t-1} + (1-\beta_1)g_t \quad (3)$$

$$v_t = \beta_2 v_{t-1} + (1-\beta_2)g_t^2 \quad (4)$$

where θ_t = weight matrix at time step t, g_t = gradient at time step t, η = learning rate
m_t = exponentially decaying average of past gradients at time step t, and
v_t = exponentially decaying average of past squared gradients at time step t

Adam has an advantage over other optimizers by implementing an adaptive learning rate for each parameter. This learning rate (η) was tuned by including bias corrected exponentially decaying average of the past gradients $\widehat{m_t}$ and the past squared gradients $\widehat{v_t}$ term (described in equation (2)-(4)) to the learning rate). The adaptive learning rate can be described as $\frac{\eta}{\sqrt{\widehat{v_t}} + \epsilon}\widehat{m_t}$ in equation (1). Therefore, the higher the average of the past gradient is the higher the adaptive learning rate. We also implemented an early stop to the iterative optimization procedure by monitoring maximum accuracy to enhance the performance of our model (Figure S8). By plotting numbers of iteration against model loss between the training and testing set, we show that the model losses of the training and testing sets are comparable demonstrating that our model complexity is appropriate and we are not overfitting our training data (Figure S8).

Optimization Categories	Approaches	ROC-AUC
Loss Function	Mean Square Error	0.84
	Mean Absolute Error	0.75
	Binary Cross-entropy	0.79
Optimizer	SGD	0.71
	Adam	0.84
	RMSprop	0.71
Regularization	L1	0.68
	L2	0.84
	L1&L2	0.7
Early Stop	Monitor maximum testing accuracy	0.84
	Monitor minimum testing loss	0.76
	Without early stop	0.77

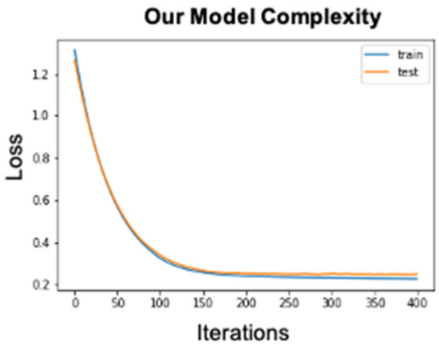

Figure S8: Mean square error loss function, Adam optimizer, L2 regularization with $\lambda = 0.01$ and implementation of early stop by monitoring testing accuracy yield the highest model performance. Plotting model loss vs number of iteration for both training set and testing set also demonstrated that model complexity was not too high or low after implementing all the optimization parameters.

References

1. Hubert M, Vandervieren E. An adjusted boxplot for skewed distributions. *Comput Stat Data Anal*. 2008;52:5186-5201. doi:10.1016/j.csda.2007.11.008
2. Weisberg S. Yeo-Johnson Power Transformations. 2001;(2).
3. Chren WA. One-Hot Residue Coding for Low Delay-Power Product CMOS Design. 1998;45(3):303-313.
4. Chen X, Jeong JC. Enhanced Recursive Feature Elimination. 2007:429-435. doi:10.1109/ICMLA.2007.35
5. Chen T, Guestrin C. XGBoost : A Scalable Tree Boosting System. 2016.
6. Cortes C, Vapnik V. Support-Vector Networks. 1995;297:273-297.
7. Breiman L. Random Forests. *Stat Dep Univeristy Calif Berkeley*. 2001:1-33.
8. Geurts P, Ernst D, Wehenkel L. Extremely randomized trees. 2006;(June 2005).
9. Freund Y, Schapire RE, Hill M. Experiments with a New Boosting Algorithm. 1996
10. Kingma DP, Ba JL. ADAM: A Method For Stochastic Optimization. *ICLR*. 2015:1-15.
11. Friedman BJH. Greedy Function Approximation: A Gradient Boosting Machine. *Ann Stat*. 2001;29(5):1189-1232.
12. Denoyer L, Gallinari P. Deep Sequential Neural Network. *arXiv*. 2014:1-9
